# Emergence of infectious bronchitis virus genotype GI-24 and GI-16 along with predominant circulation of genotype GI-1 in commercial poultry in Bangladesh

**DOI:** 10.1016/j.psj.2026.106980

**Published:** 2026-04-20

**Authors:** Mohosin Kabir, MostShahana Akter, Md. Riabbel Hossain, Md. Mohi Uddin, Adam Jbenyeni, Gwenaëlle Dauphin, EmdadulHaque Chowdhury, Rokshana Parvin

**Affiliations:** a Department of Pathology, Faculty of Veterinary Science, Bangladesh Agricultural University, Mymensingh 2202, Bangladesh; b CEVA Santé Animale, 33500 Libourne, France

**Keywords:** Infectious bronchitis virus, S1 gene, Molecular epidemiology, Vaccine escape, Bangladesh

## Abstract

Persistent Infectious bronchitis virus (IBV) outbreaks in Bangladesh, indicate inadequate vaccination strategy, low vaccination quality or inconsistent coverage of all the poultry production in terms of IBV. This study investigated the prevalence and genetic diversity of IBV in commercial poultry farms in Bangladesh. A total of 390 samples from commercial broiler and layer flocks (July 2022-June 2025) were screened by RT-qPCR, with selected positives subjected to in ovo isolation and S1 gene sequencing for phylogenetic analysis. IBV was the most prevalent respiratory pathogen, detected in 26.67% of samples and often co-circulating with others. Phylogenetic analysis of the S1 gene revealed the co-circulation of three distinct IBV genotypes: GI-1, GI-16, and GI-24. The GI-1 (Mass-type) genotype was predominant and exhibited high nucleotide identity (99.8–100%) with commonly used vaccine strains, including Ma5, H120, and B-48. Additionally, two other genotypes were identified for the first time in Bangladesh: GI-24, closely related to nephropathogenic Indian strains, and GI-16 (Q1-like), a highly transmissible variant widely reported in Europe and Asia. Pairwise genetic distance analysis demonstrated measurable divergence between vaccine strains and circulating field isolates, potentially explaining continued viral circulation and disease occurrence in vaccinated flocks. Notable genetic variation was observed within S1 hypervariable regions, including mutations affecting predicted neutralizing B-cell epitopes, suggesting antigenic divergence that may contribute to reduced vaccine-induced protection against emerging genotypes. These findings suggest IBV introduction events in Bangladesh and support continuous molecular surveillance in order to test the cross-protection capability of the commercially available vaccine strains.

## Introduction

Infectious Bronchitis (IB) is a highly contagious and economically significant viral disease of poultry, particularly in commercial chickens (Ramakrishnan and Kappala, 2019; Rafique et al., 2024). The disease is caused by the Infectious Bronchitis Virus (IBV), a positive-sense, single-stranded, enveloped RNA virus that belongs to the avian coronaviruses, members of the genus *Gammacoronavirus*, within the family Coronaviridae, and order Nidovirales (Woo et al., 2023). The disease first appeared in the 1930s, posing a major threat to global poultry production due to its wide tissue tropism, high transmissibility, and the emergence of numerous serotypes and genotypes (M. Najimudeen et al., 2020; Rafique et al., 2024). It primarily replicates in the tracheal mucosa, but nephropathogenic and reproductive variants have also been reported, leading to complex clinical presentations and substantial economic losses (Bhuiyan et al., 2021; Khan et al., 2025).

The genome of IBV consists of spike glycoprotein (S), membrane protein (M), envelope protein (E), and nucleocapsid protein (N). Spike glycoprotein (S) consists of S1 and S2 subunits (Parvin et al., 2021; Junnu and Pohuang, 2022). The S1 subunit is responsible for viral attachment and induction of neutralizing antibodies, and the S2 subunit is responsible for fusion between the viral and host cell membranes (Eldemery et al., 2017). Genetic variability is particularly pronounced in the S1 subunit (Valastro et al., 2016), which forms a distinguishing feature, extensive genetic and antigenic diversity, resulting from the high mutation rate of RNA viruses and frequent recombination events during replication (Marandino et al., 2019).

From 1931 to the recent IBV identification, there are nine genotypes and 39 lineages, namely GI-1 to GI-30, GII-1, GII-2, and GIII-1 to GIX-1 (Hou et al., 2020; Rafique et al., 2024; Xiong et al., 2024). Among these, GI-1 (Massachusetts type) remains the most prevalent strain of many live and inactivated vaccines used worldwide (Sheng et al., 2020; Alhafufi et al., 2025). Despite widespread vaccination, outbreaks associated with GI-1 and GI-1-like viruses continue to occur, highlighting the persistence and adaptability of this lineage under vaccine pressure (Abozeid, 2023). Moreover, the extensive use of Mass-type vaccines has been suggested to contribute to viral evolution and the emergence of new variants through recombination (Ball et al., 2016).

In recent years, emerging IBV lineages, including GI-24, which has been reported primarily from South Asia (Valastro et al., 2016; Saleem et al., 2024). Although the epidemiological and pathogenic characteristics of GI-24 are not yet fully understood, its detection raises concerns regarding vaccine mismatch (Guzmán et al., 2025). The emergence of novel genotypes emphasizes the dynamic nature of IBV evolution and the necessity for continuous surveillance.

In Bangladesh, the poultry industry is a rapidly expanding sector for meeting the demand for high-quality protein of its large population (Hamid et al., 2016). However, the sector faces significant challenges from infectious diseases, including IB (Parvin et al., 2021; Salles et al., 2025). Previous studies in Bangladesh have reported the circulation of several IBV genotypes, including Mass-like (GI-1), 4/91-like (GI-13), and QX-like (GI-19) strains in both broiler and layer flocks, despite routine vaccination programs (Parvin et al., 2021, 2024).

Continuous monitoring of field outbreaks is a cornerstone for the detection and evaluation of newly emerging IBV genotypes. It is equally important to determine whether existing vaccination strategies remain fully effective against the genetically diverse and continuously evolving IBV population circulating in the country. For these reasons, comprehensive investigation of the genetic characteristics of newly emerging genotypes, as well as the prevalence of currently circulating genotypes in commercial poultry, is essential. In addition, identification of key regions of genetic variation within the viral spike (S) protein is critical for understanding IBV evolution and for the development of well-matched and stable vaccines. Therefore, the study aimed to investigate the prevalence, genetic diversity, and evolutionary characteristics of IBV circulating in commercial poultry flocks in Bangladesh.

## Materials and methods

### Sample collection

A total of 390 samples were collected from commercial poultry flocks suspected of being infected with infectious bronchitis virus (IBV), exhibiting clinical signs of respiratory distress, including gasping, coughing, and difficulty breathing, across Bangladesh between 2022 and 2025. The samples were obtained randomly, including broilers (*n* = 220), layers (*n* = 100), broiler breeders (*n* = 20), and sonali chickens (*n* = 50), distributed across eight major poultry-producing regions: Dhaka (*n* = 92), Mymensingh (*n* = 98), Rajshahi (*n* = 50), Rangpur (*n* = 40), Chattogram (*n* = 30), Sylhet (*n* = 20), Khulna (*n* = 30), and Barishal (*n* = 30). Samples consisted of oropharyngeal and cloacal swabs, as well as tissue samples (trachea, lungs, and kidneys) collected from clinically suspected birds.

Sample collection was performed using sterile swabs and instruments under strict aseptic conditions. Each sample was immediately placed into viral transport medium (VTM) supplemented with antibiotics (penicillin, 10,000 IU/mL; streptomycin, 10 mg/mL) to inhibit bacterial contamination. Samples were transported to the laboratory and stored at −80°C until further processing.

### RNA extraction and detection by RT-qPCR

Viral RNA was extracted from 200 μL of swab suspension or clarified tissue homogenate using the GeneJET^TM^ RNA Purification Kit (Thermo Fisher Scientific, USA), following the manufacturer’s instructions. RNA concentration and purity were assessed using a NanoDrop One/One^c^ Microvolume UV-Vis Spectrophotometer (Thermo Fisher Scientific, USA). Detection of avian Gammacoronavirus (AvCoV) was performed using a single-step reverse transcription quantitative PCR (RT-qPCR) targeting the 5′ untranslated region (5′-UTR) of the IBV genome as described by Callison et al., 2006. The assay was carried out using the Luna Universal One-Step RT-qPCR Kit (New England Biolabs Inc., USA) with SYBR Green chemistry.

Each RT-qPCR reaction was performed in a final volume of 12.5 μL, containing 2.5 μL RNA template, 5 μL of 2 × RT-qPCR SYBR reaction mix, 0.5 μL Luna WarmStart RT Enzyme Mix, 2 μL of IBV-specific primer mix (10 pmol/μL each), and 2.5 μL nuclease-free water. Thermal cycling conditions consisted of reverse transcription at 55°C for 10 min, initial denaturation at 95°C for 1 min, followed by 45 cycles of denaturation at 95°C for 10 s and annealing/extension at 60°C for 30 s. Fluorescence data were collected during the annealing/extension step. The primers used in detection are given in Table 1.

**Table 1:** List of primers used for the molecular detection and amplification of target genes of infectious bronchitis in commercial poultry.

Target	PCR Approach	Primes	References
Avian corona virus Combination of untranslated region and polymerase gene	Real time SYBR green based	SYBR-Green based RT-qPCR IBVGU391: GCT TTT GAG CCT AGC GTT IBV GL533: GCC ATG TTG TCA CTG TCT ATT Av-CoV_Fw: GGT TGG GAT TAT CCW AAR TGT G Av-CoV_Rv: TGY TGT GAR CAA AAY TCR TG	Chamings et al. (2018)
S1 gene of IBV	Conventional	Forward primer: GCCAGTTGTTAATTTGAAAAC Reverse primer: TAATAACCACTCTGAGCTCT	Pohuang et al. (2011)

### Virus isolation

Virus isolation was conducted using 11-day-old specific pathogen-free (SPF) embryonated chicken eggs (ECEs). Tissue homogenates and swab suspensions were clarified by centrifugation at 6,000 rpm for 15 min, followed by filtration through 0.45 μm syringe filters. A volume of 200 μL of the filtered supernatant was inoculated into the allantoic cavity of each ECE. Inoculated eggs were incubated at 37°C and monitored daily for embryo viability. Embryo mortality occurring within 24 h post-inoculation was considered non-specific and excluded from analysis. After 96 h, allantoic fluids were harvested aseptically and stored at −80°C for further molecular analysis.

### Reverse transcription and PCR amplification

Molecular confirmation and genotyping of IBV were performed by amplification of the partial S1 gene as described by Pohuang et al., 2011 (Table 1) using the SuperScript™ III One-Step RT-PCR System with Platinum™ Taq DNA Polymerase (Invitrogen, Thermo Fisher Scientific, USA). RT-PCR reactions were carried out in a final volume of 25 μL, comprising 12.5 μL of 2 × reaction mix, 0.5 μL SuperScript™ III RT/Platinum™ Taq mix, 1 μL each of forward and reverse primers, 2.5 μL RNA template, and 7.5 μL nuclease-free water.

The RT-PCR cycling conditions included reverse transcription at 48°C for 30 min, initial denaturation at 94°C for 1 min, followed by 39 cycles of denaturation at 94°C for 15 s, annealing at 53°C for 30 s, and extension at 68°C for 40 s, with a final extension at 68°C for 5 min. Amplified products were resolved on 1.5% agarose gels, stained with ethidium bromide, and visualized under UV illumination.

### Sequence and phylogenetic analysis

PCR products of specifically the eighteen positive isolates selected for full S1 gene amplification and sequencing were purified using the ExoSAP-IT PCR Product Cleanup Kit (Affymetrix Inc., Cleveland, OH, USA) by incubation at 37°C for 15 min, followed by enzyme inactivation at 80°C for 15 min. Cycle sequencing was performed using the ABI BigDye Terminator v3.1 Cycle Sequencing Kit (Applied Biosystems, Foster City, CA, USA) according to the manufacturer’s instructions. The sequencing reactions were analyzed on an ABI 3500 XL Genetic Analyzer (Applied Biosystems, Foster City, CA, USA).

The obtained nucleotide sequences of the positive IBV isolates were submitted to GenBank, under their corresponding accession numbers PX938991-PX939006. Multiple sequence alignments of the partial S1 gene sequences obtained together with the reference strains provided by Valastro et al. (2016) and the related published strains of IBV from the GenBank database were performed using MAFFT version 7 online tool (Katoh et al., 2019). Finally, the phylogenetic tree was constructed using the maximum likelihood method employing the GTR +*G* + *I* + *F* model and SH-like aLRT method for branch support via the NGPhylogeny online tool (Lemoine et al., 2019). The tree was annotated using iTOL.

### Amino acid variation and hypervariable region (HVR) analysis

Deduced amino acid sequences of the S1 protein were analyzed to identify mutations within known hypervariable regions (HVRs), particularly HVR1–3. Amino acid substitutions at key positions (e.g., residues 38, 64, 69, 121, 126, 181, 273, and 296) were identified through multiple sequence alignment and compared with vaccine strains (Ma5, H120) and global reference strains. Pairwise genetic distance analysis was performed to assess divergence between field isolates and vaccine strains.

### Prediction of linear B-cell neutralizing epitopes

Linear B-cell epitope prediction was conducted on deduced S1 amino acid sequences using established in silico epitope prediction tools (e.g., BepiPred). Particular attention was given to previously reported neutralizing epitope regions located at amino acid positions 87–93 and 412–418. Predicted epitope sequences were compared across genotypes (GI-1, GI-16, and GI-24) to assess sequence variability and potential implications for antigenicity and cross-protective immunity.

### Statistical analysis

Data was compiled and managed in Microsoft Excel (Microsoft Corp., Redmond, WA, USA) and analyzed using GraphPad Prism 10.4.2. Descriptive statistics were used to summarize the distribution of samples, and IBV prevalence was expressed as percentages of RT-qPCR-positive samples relative to the total number tested. Differences in IBV prevalence among poultry types and geographic regions were assessed using the chi-square (χ²) test. The association between viral load (categorized based on Ct values) and clinical severity scores was evaluated using Spearman’s rank correlation coefficient. All statistical analyses were performed using two-tailed tests, and differences were considered significant at *p* < 0.05. Molecular divergence estimation among infectious bronchitis virus (IBV) sequences were based on the number of base substitutions per site between sequence pairs. Analyses were performed using the Maximum Composite Likelihood model (Tamura et al., 2004) with the pairwise deletion option applied to ambiguous positions, resulting in a final dataset of 3,507 nucleotide positions. Evolutionary analyses were conducted in MEGA 12 (Kumar et al., 2024).

## Results

### Sample screening and IBV detection by RT-qPCR

Out of the 390 samples collected from commercial poultry flocks across Bangladesh, 104 samples tested positive for infectious bronchitis (IB) by RT-qPCR. Out of 100-layer flocks investigated, 16 were positive. Among broiler flocks, 79 out of 220 tested positive, while 3 out of 20 broiler breeder flocks and 15 out of 50 Sonali flocks were positive, corresponding to positivity rates of 16%, 31.82%, 15%, and 30%, respectively. Overall, 104 of the 390 flocks examined were confirmed positive, indicating nationwide distribution of IBV during the study period (2022–2025).

### Geographical distribution of IBV in Bangladesh

**Fig. 1:**
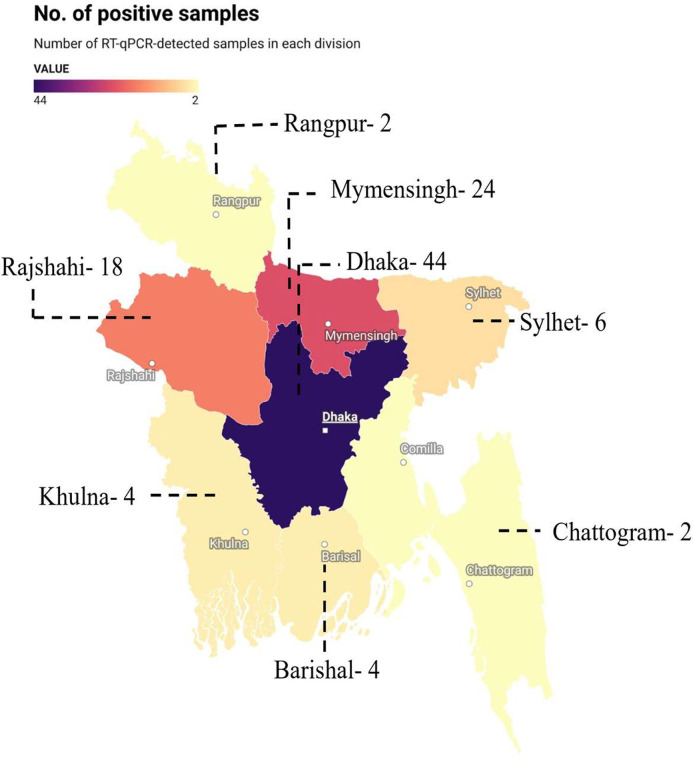
Regional occurrence of IBV across different regions of Bangladesh. The map shows the number of positive IBV samples across different administrative divisions. The figure was created by using the website https://www.datawrapper.de.

IBV-positive samples were distributed across multiple administrative divisions, including Dhaka, Mymensingh, Rajshahi, Rangpur, Chattogram, Sylhet, Khulna, and Barishal. Dhaka division showed the highest positive rate, with 44 of 92 samples testing positive (47.8%). Mymensingh recorded 24 positives from 98 samples (24.5%), followed by Rajshahi with 18 positives out of 50 samples (36.0%). Sylhet reported 6 positive samples from 20 (30.0%), while Rangpur showed the lowest prevalence, with 2 positives from 40 samples (5.0%). Chattogram had 2 positive cases from 30 samples (6.7%), and both Khulna and Barisal each recorded 4 positives from 30 samples (13.3%) (Fig. 1).

### Correlation of viral loads with clinical signs

**Fig. 2:**
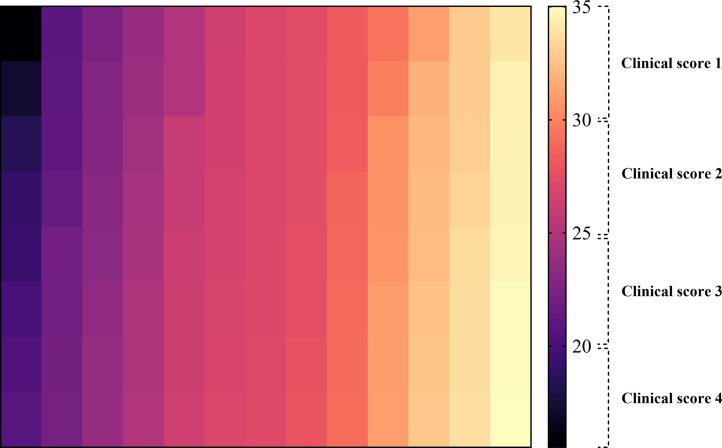
The heat map illustrates the correlation between viral load and clinical severity. Darker color intensities represent higher viral loads, which correspond to elevated clinical scores. In contrast, progressive lighter shades indicate lower viral loads associated with mild or minimal clinical manifestations. The figure was created using the GraphPad Prism 10.4.2.

RT-qPCR analysis revealed substantial variation in viral load among the IBV-positive samples, as reflected by the range of Ct values. However, the Ct value may vary due to the sample collection time point and the infection phase, flocks exhibiting more severe respiratory manifestations, including tracheal rales, nasal discharge, coughing, and reduced egg production in layers, consistently showed lower Ct values, indicating higher viral loads. In contrast, samples from flocks with mild or minimal clinical signs showed higher Ct values, indicating a lower viral burden (Table 2 & Fig. 2).

**Table 2:** Correlation of viral load obtained at RT-qPCR with clinical and gross pathological changes.

Range of CT value	Viral load	No. of samples	Clinical signs and gross lesions	Clinical Score	Viral copy number per mL
Less than 20	Very high viral load	*6	High to moderate fever, severe respiratory distress (rales, tracheal exudate, coughing), higher morbidity at flock level, exudative & hemorrhagic trachea, congested & consolidated lungs, swollen kidney, urate deposition (nephropathogenic effects), sharp drop in egg production (layers)	4	Greater than 10^6^
Between 21-25	High viral load	*28	Moderate to higher morbidity at the flock level, moderate to severe respiratory distress, and persistent coughing. Hemorrhage on the trachea, congested and consolidated lungs, swollen kidney.	3	Between 10^5^-10^6^
Between 26-30	Moderate viral load	*40	Low-grade fever, sneezing, nasal discharge, subclinical or early infection, mild hemorrhage, and congestion in the lungs.	2	Between 10^3^-10^5^
Greater than 30-35	low viral load	*30	Mostly asymptomatic, showing inappetence and drowsiness, or a history of late infection stage (virus clearance phase)	1	Between 10^2^-10^3^

*Evidence of coinfection in 62/104 positive flocks either with AIV or NDV.

### Mixed infection of IBV with other viruses

Out of the 104 IBV-positive samples, 62 (59.6%) were co-infected with other common respiratory viruses, as determined by RT-qPCR targeting avian influenza virus (AIV) and Newcastle disease virus (NDV). Among the co-infections, IBV with NDV was the most frequent combination, detected in 39 samples. Co-infection with AIV was also observed, including concurrent detection of IBV with H9 subtype in 19 samples and with H5 subtype in 4 samples. Furthermore, the severity of clinical disease may also be influenced by the presence of co-infections (e.g., bacterial or viral respiratory pathogens) and/or incomplete or improper vaccination programs, which could contribute to enhanced clinical expression despite variable viral loads. Notably, 12 out of 34 flocks with the lowest Ct values (highest viral loads) were co-infected with AIV or NDV, indicating that mixed infections may contribute to enhanced respiratory disease severity and prolonged viral replication (Table 2). Interestingly, co-infected flocks exhibited comparatively higher mortality and higher viral loads for at least one of the detected respiratory pathogens. A notable pattern was observed in several co-infected cases: when IBV showed the highest viral load (lower Ct value), the co-detected agent (e.g., AIV or NDV) often exhibited a relatively lower viral load (higher Ct value), and vice versa.

Overall, these findings suggest that IBV viral load is positively associated with clinical severity, while co-infections and incomplete vaccination programs may act as important aggravating factors influencing the outcome of IBV infection under field conditions.

### Virus isolation in embryonated chicken eggs

**Fig. 3:**
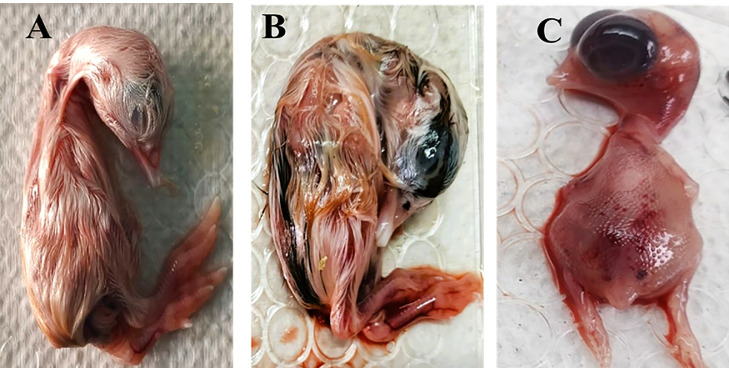
Embryonic changes are associated with infectious bronchitis virus infection. (A) Uninfected control embryo showing normal development. (B, C) IBV-infected embryos exhibit stunting, curling, dwarfism, and subcutaneous hemorrhage.

Among 10 selected strains, 3 strains were successfully propagated in 11-day-old embryonated chicken eggs (ECEs). Inoculated embryos showed characteristic IBV-associated changes, including embryo dwarfing, curling, subcutaneous hemorrhage (Fig. 3), and death after 24 h post-inoculation, while early embryo mortality (<24 h) was excluded as non-specific. Following the 96-hour incubation for each passage, the allantoic fluid was successfully harvested and confirmed positive for IBV by molecular assays. Lower isolation success may be attributed to the fact that some field samples contained low levels of infectious virus despite being RT-qPCR positive, particularly when collected at later stages of infection or after partial immune clearance. In addition, the presence of maternal or vaccine-induced antibodies may reduce virus viability and interfere with efficient replication in embryonated chicken eggs. Furthermore, certain IBV field strains may exhibit poor adaptation to propagation in embryonated eggs, which could also contribute to reduced isolation efficiency.

### Amplification of the IBV S1 gene

**Fig. 4:**
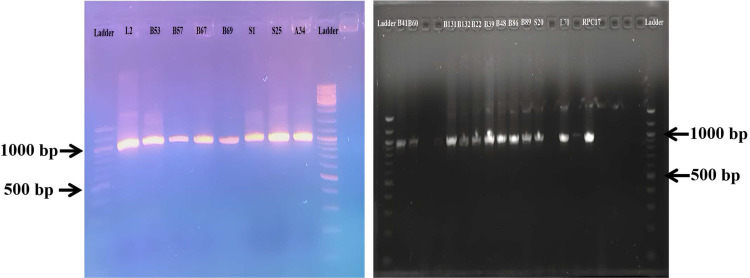
Agarose gel electrophoresis showing amplification of the S1 gene of infectious bronchitis virus (IBV). A specific amplicon of approximately 1,000 bp is observed in positive samples when compared with the molecular weight ladder.

The successful amplification of the partial S1 gene fragment of Infectious Bronchitis Virus (IBV) isolates was confirmed by using one-step RT-PCR. Agarose gel electrophoresis in a 1.5% agarose gel revealed bands of around 1000 bp, confirming successful amplification of the target region (Fig. 4). These amplicons were subsequently processed for sequencing and genetic characterization.

### Sequence analysis and genotype identification

Partial sequencing of the S1 gene, encompassing hypervariable regions (HVRs) 1 and 2 (312 nucleotides) and HVR3 (342 nucleotides), was performed, as these regions are widely used for molecular classification of IBV. A total of 18 IBV-positive samples yielded successful S1 gene amplification and were included for sequence analysis. Among these, 16 high-quality sequences (S1, B22, B39, B41, S20, B48, B53, S25, B60, B67, B69, L70, B86, B89, B131, and B132) were retained for genotypic classification. Comparative sequence analysis revealed the circulation of three distinct IBV genotypes in commercial poultry flocks in Bangladesh during the study period. Most isolates belonged to genotype GI-1 (Massachusetts-like lineage; *n* = 13), followed by GI-24 (*n* = 2) and GI-16 (Q1-like lineage; *n* = 1). Within the GI-1 lineage, a major cluster of 13 isolates (S1, S20, B22, B39, B41, B48, L70, B53, B67, B86, B89, B131, and B132) was identified. Eleven of these isolates exhibited nucleotide identity (98.6-100%) with commonly used Mass-type vaccine strains, like Ma5, B-48, and H-120 (Table 3), suggesting the circulation of vaccine-like strains in the field. Notably, two GI-1 isolates (B53 and B67) showed 99.88% nucleotide identity with an older Chinese field strain (ck/CH/LHB/111232/2011), indicating the simultaneous circulation of both vaccine-derived and field-origin GI-1 viruses in Bangladesh. This detection could be also the recovery of the GI-1 vaccine administered to the birds as most vaccinated flocks are using this strain. Two isolates (S25 and B60) clustered within the GI-24 lineage, exhibiting approximately 96.86% nucleotide similarity to a nephropathogenic Indian strain (IB/MZ/IND/2/2015), confirming the presence of this genetically distinct South Asian lineage. In addition, one isolate (B69) grouped within the GI-16 (Q1-like) lineage and showed 100% nucleotide identity with an Italian field strain (gammaCoV/Ck/Italy/I2022/2013), providing the first molecular evidence of GI-16 circulation in Bangladesh.

**Table 3:** List of sequenced IBV samples showing sample IDs, RT-qPCR Ct values, closest matching reference strains, percentage sequence similarity, and assigned genotypes.

SL No	Sample ID	CT value	Highest similarities	Similarity index	Genotypes
1	S1/Sonali/2022	26.23	KY626045 strain Ma5 (Nobilis vaccine strains)	99.05%	GI-1
2	B22/Broiler/2022	20.93	FJ888351_H120_The Netherlands_1960 (live-attenuated vaccine)	99.8%	GI-1
3	B39/Broiler/2022	18.37	B-48 vaccine strain (CEVAC® MASS L LIVE VACCINE)	100%	GI-1
4	B41/Broiler/2022	23.46	B-48 vaccine strain (CEVAC® MASS L LIVE VACCINE)	100%	GI-1
5	S20/Sonali/2022	26.38	FJ888351_H120_TheNetherlands_1960 (live-attenuated vaccine)	99.8%	GI-1
6	B48/Broiler/2022	19.95	FJ888351_H120_TheNetherlands_1960 (live-attenuated vaccine)	100%	GI-1
7	B53/Broiler/2023	22.55	ck/CH/LHB/111232/2011 (Chinese field strain)	99.88%	GI-1
8	S25/Sonali/2023	22.1	IB/MZ/IND/2/2015 (Indian field strain)	96.86%	GI-24
9	B67/Broiler/2023	21.4	ck/CH/LHB/111232/2011 (Chinese field strain)	99.88%	GI-1
10	B60/Broiler/2023	26.01	IB/MZ/IND/2/2015 (Indian field strain)	96.86%	GI-24
11	B69/Broiler/2023	20.68	gammaCoV/Ck/Italy/I2022/2013 (Italian field strain)	100%	GI-16 (Q1-like)
12	L70/Layer/2023	27.93	FJ888351_H120_TheNetherlands_1960 (live-attenuated vaccine)	99.8%	GI-1
13	B86/Broiler/2023	27.01	B-48 vaccine strain (CEVAC® MASS L LIVE VACCINE)	100%	GI-1
14	B89/Broiler/2023	27.38	B-48 vaccine strain (CEVAC® MASS L LIVE VACCINE)	100%	GI-1
15	B131/Broiler/2024	23.84	B-48 vaccine strain (CEVAC® MASS L LIVE VACCINE)	100%	GI-1
16	B132/Broiler/2024	26.52	B-48 vaccine strain (CEVAC® MASS L LIVE VACCINE)	100%	GI-1

### Phylogenetic and nucleotide distance analysis

**Fig. 5:**
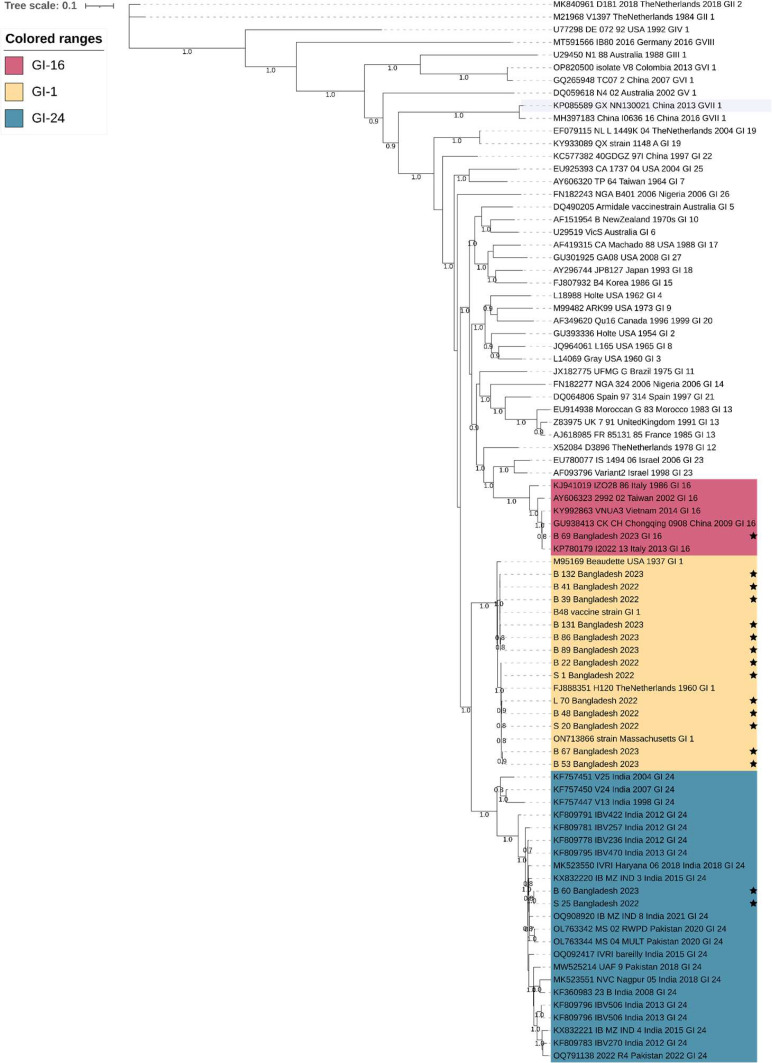
Phylogenetic tree based on partial S1 gene sequences of 16 Bangladeshi infectious bronchitis virus (IBV) isolates together with 91 reference sequences retrieved from GenBank. The tree was constructed using MAFFT version 7 online tool (Katoh et al., 2019). The phylogenetic tree was constructed using the maximum likelihood method employing the GTR +*G* + *I* + *F* model and SH-like aLRT method for branch support via the NGPhylogeny online tool (Lemoine et al., 2019). The tree was annotated using iTOL. Bootstrap values are shown at the corresponding nodes, and GenBank accession numbers are indicated alongside strain names. Colors represent the three different genotypes as mentioned in the color range box. Star mark denotes Bangladeshi strains sequenced in this study.

Maximum likelihood (ML) phylogenetic trees were constructed based on partial S1 gene sequences using a reference sub-sampled dataset of 91 global IBV strains. Phylogenetic inference demonstrated the co-circulation of three IBV genotypes (GI-1, GI-24, and GI-16) in Bangladesh, as shown in Fig. 5. GI-1 (Mass-type) was identified as the predominant genotype, consistent with its global distribution and extensive use of vaccines. The GI-24 isolates formed a distinct and well-supported clade closely related to Indian reference strains, while the GI-16 isolate clustered firmly with Q1-like strains previously reported from Europe and Asia. These findings indicate the introduction of non-Mass-type IBV lineages into the Bangladeshi poultry population.

**Fig. 6:**
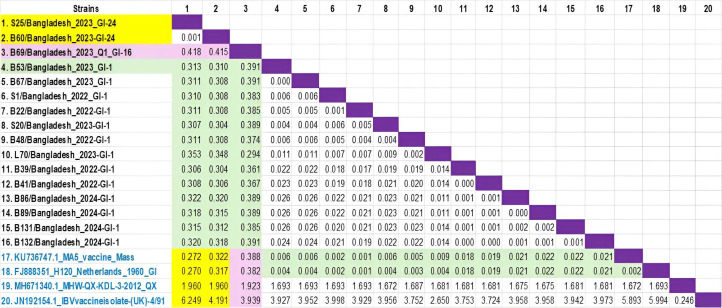
Estimates of evolutionary divergence among infectious bronchitis virus (IBV) sequences based on the number of base substitutions per site between sequence pairs. Analyses were performed using the Maximum Composite Likelihood model (Tamura et al., 2004) with the pairwise deletion option applied to ambiguous positions, resulting in a final dataset of 3,507 nucleotide positions. Evolutionary analyses were conducted in MEGA 12 (Kumar et al., 2024). Yellow shading indicates measurable genetic divergence between the newly introduced GI-24 genotype with reference vaccine strains, pink denotes genetic divergence between GI-16 genotype with reference vaccine strains, whereas green denotes negligible genetic variation among strains of GI-1field and vaccine strains.

Pairwise evolutionary distances between field isolates and commonly used vaccine strains were estimated using the Maximum Composite Likelihood model (Fig. 6). The analysis revealed substantial genetic divergence between several field strains and vaccine strains. The newly emerged genotype GI-24 (B57 and B60) showed a significant distance from the field strains of GI-16 and GI-1 (Mass type) characterized in the current study. Similarly, GI-24 also demonstrated a significant genetic divergence from the reference vaccine strains like MA5, H120, QX-KDL, and 4/91 (Fig. 6). Interestingly, the studied GI-1 field isolates exhibited measurable genetic variation among field and vaccine strains, which may contribute to differences in pathogenicity and clinical outcomes observed in the field.

Overall, the results demonstrate the concurrent circulation of Mass-type vaccine strains (Ma5, B-48, and H-120) alongside genetically distinct field strains of Chinese (GI-1), Indian (GI-24), and Italian (GI-16) origin during the study period. The global distribution patterns of these genotypes further support their epidemiological relevance: GI-1 is globally widespread; GI-16 originated in China and has spread across Asia, Europe, South America, and Africa; while GI-24 has been primarily reported from India and Pakistan and is now confirmed to be emerging in Bangladesh.

### Genetic variation at HVRS and neutralizing epitope site

The present study further focused on the spike (S1) protein, which typically comprises 540–543 amino acids and contains hypervariable regions (HVRs) that exhibit the highest degree of genetic variability within the IBV genome. Several key amino acid substitutions were identified within the HVRs at positions 38, 64, 69, 121, 126, 181, 273, and 296. These residues are considered critical determinants of cell tropism, antigenicity, and vaccine efficacy, and mutations at these sites have been associated with the emergence of new serotypes and challenges in disease control (Farooq et al., 2024). Notably, substitutions such as D38N, G64E, and I69T were detected within HVRs 1–2.

Six IBV strains (B39, B41, B86, B89, B131, and B132) belonging to genotype GI-1 (Mass-like) exhibited D38N and I69T substitutions when compared with other characterized GI-1 strains, including the vaccine strains Ma5 and H120. These amino acid changes suggest possible immune escape driven by vaccine-induced selective pressure. In addition, strain B131 harbored a G64E substitution, a mutation previously associated with altered tissue tropism and increased virulence.

The two newly identified strains (S25 and B60) classified under genotype GI-24 also carried D38N and G64E substitutions, indicating potential antigenic drift that may contribute to vaccine escape and the emergence of new serotypes or altered tissue tropism. In contrast, the reference GI-24 strain (IB-MZ-IND) possessed a D38T substitution, distinct from the Bangladeshi GI-24 strains. However, further experimental studies are required to determine the biological significance of these mutations within GI-24 viruses.

Genotype GI-16 appeared relatively conserved across the analyzed isolates. Similarly, genotypes GI-13 and GI-19, detected in our earlier studies (Zeng et al., 2025), remained conserved at these key positions (Table 4). Moreover, amino acid residues at positions 273 and 296 within HVR3 were conserved within their respective genotypes, suggesting functional constraints at these sites.

**Table 4:** Amino acid comparison of Bangladeshi field strains within their respective groups of genotypes and with selected vaccine strains.

Genotypes	Strains	HVR1-2						HVR3	
		38	43	63	64	69	181	273	296
GI-24 (Indian variant)	ASV64799.1 IB-MZ-IND G-24	T	H	^∆^	**E**	I	S	L	L
	S25/Bangladesh_2023_GI-24	**N**	H	^∆^	**E**	I	S	L	L
	B60/Bangladesh_2023-GI-24	**N**	H	^∆^	**E**	I	S	L	L
GI-16 (Q1-like)	B69/Bangladesh_2023_Q1_GI-16	D	H	S	Q	I	S	L	-
GI-1 (Mass like)	B53/Bangladesh_2023_GI-1	D	H	S	G	I	S	L	T
	B67/Bangladesh_2023_GI-1	D	H	S	G	I	S	L	T
	S1/Bangladesh_2022_GI-1	D	H	S	G	I	S	L	T
	B22/Bangladesh_2022-GI-1	D	H	S	G	I	S	L	T
	S20/Bangladesh_2023-GI-1	D	H	S	G	I	S	L	T
	B48/Bangladesh_2022-GI-1	D	H	S	G	I	S	L	T
	L70/Bangladesh_2023-GI-1	D	H	S	G	I	S	L	T
	B39/Bangladesh_2022-GI-1	**N**	H	S	G	**T**	S	L	T
	B41/Bangladesh_2022-GI-1	**N**	H	S	G	**T**	S	L	T
	B86/Bangladesh_2024-GI-1	**N**	H	S	G	**T**	S	L	T
	B89/Bangladesh_2024-GI-1	**N**	H	S	G	**T**	S	L	T
	B131/Bangladesh_2024-GI-1	**N**	H	S	**E**	**T**	S	L	T
	B132/Bangladesh_2024-GI-1	**N**	H	S	G	**T**	S	L	T
	KU736747.1 IBV MA5 vaccine	D	H	S	G	I	S	L	T
	FJ888351_H120_Netherlands_1960_GI-1 (Vaccine)	D	H	S	C	I	S	L	T
GI-19 (QX-type)	AFM46264.1 IBV_S1 QX-like	P	L	A	S	G	N	T	Y
GI-13 (4/91 type)	KX107649.1_IBV_ CK/CH/FJ/PT1301 S1_4/91	S	L	V	S	G	N	T	Y

Foot note: - Missing coverage; ∆ Deletion; bold indicates potential mutation.

Furthermore, neutralizing epitope sites were predicted within the hypervariable regions (HVRs) of the S1 gene that usually showed marked differences among multiple IBV genotypes (Niesters et al., 1987). In particular, two linear B-cell epitopes located on the S1 subunit at amino acid positions 87–93 (highly variable) and 412–418 (relatively conserved) were observed in the IBV strains circulating in Bangladesh. These epitopes play a critical role in the induction of neutralizing antibodies that block viral attachment to host cells (Niesters et al., 1987; Zou et al., 2015). Consequently, amino acid substitutions within these regions may alter neutralizing activity and contribute to reduced vaccine efficacy against certain strains.

The linear B-cell epitope at positions 87–93 exhibited notable sequence variation among the identified genotypes. Genotype GI-24 showed the motif ^PSLGMGW^, genotype GI-16 exhibited ^PELGMTW^, whereas genotype GI-1 carried the sequence ^PSSGMAW^. Additionally, the older genotype, like GI-19 (QX-type) and GI-13 (4/91 type) that has reported previously in Bangladesh exhibited ^PPQGMAW^. Such genotype-specific differences in this immunodominant epitope suggest limited cross-protection when vaccines derived from one genotype are used against heterologous genotypes. In contrast, the epitope at positions 412–418 remained relatively conserved across genotypes, indicating a potentially important functional role and a possible target for broader protective immunity.

## Discussion

Infectious Bronchitis virus (IBV) is a threat to commercial poultry production because of its high transmissibility, genetic variety, and frequent appearance of novel variants. In the present study, molecular surveillance of IBV in commercial poultry flocks across Bangladesh between 2022 and 2025 revealed the co-circulation of multiple IBV genotypes, with a clear predominance of GI-1 (Massachusetts-type) and the first molecular detection of genotypes GI-24 and GI-16 in the country. These findings provide important insights into the altered epidemiology of IBV in Bangladesh and have significant implications for disease control strategies.

In this study, co-infection with other respiratory pathogens was identified in 59.6% (62/104) of IBV-positive samples. Therefore, disease severity appeared to be influenced not only by IBV viral load but also by the presence of concurrent respiratory infections. Co-infection with other viral respiratory pathogens, particularly avian influenza virus (AIV) and Newcastle disease virus (NDV), was frequently observed among flocks with higher IBV viral loads. Within co-infected flocks, one pathogen may dominate replication at the time of sampling, potentially reflecting viral interference, competition for susceptible host cells, or differences in timing and disease progression. Such dynamics may contribute to increased clinical severity and mortality in co-infected flocks compared to single infections, emphasizing the importance of considering multi-pathogen interactions in respiratory disease outbreaks.

The predominant circulation of GI-1 (Mass-type) observed in this study is consistent with reports from many regions worldwide, where Mass-type viruses continue to circulate despite extensive vaccination programs (Jackwood et al., 2012; Marchenko et al., 2022). In Bangladesh, live vaccines based on Mass-type genotypes such as Ma5, H120, and B-48 are widely used, which might be a cause for the continuous detection of GI-1 viruses. Also important to mention that the primers used for the partial S1 amplification may have higher sensitive to GI-1 versus other field viruses in case of concomitant presence of vaccine GI-1 and field IBV which may bias the sequencing result mainly from samples for which IBV was not isolated. The very high nucleotide identity (99.8–100%) between most GI-1 field isolates and vaccine strains strongly suggests the circulation of vaccine-like viruses, possibly arising from vaccine virus persistence, reversion to virulence, or bird-to-bird transmission under field conditions. Similar findings have been reported in other countries, where Mass-type vaccine strains were frequently recovered from vaccinated flocks (Sjaak de Wit et al., 2011; Franzo et al., 2019; Rohaim et al., 2020). Notably, this study also identified GI-1 isolates closely related to an older Chinese field strain, indicating that not all GI-1 viruses detected were vaccine-derived. The co-circulation of vaccine-like and field-origin GI-1 strains highlights the complex IBV population structure shaped by vaccination pressure, viral evolution, and potential introduction through poultry trade or movement of birds and poultry products (Promkuntod, 2016; Franzo et al., 2025). Such coexistence may facilitate recombination, increasing viral diversity and complicating disease control.

This study also first detected the genotype GI-24 in Bangladesh. GI-24 has been primarily reported from India and Pakistan and is considered a regionally emerging South Asian lineage (Valastro et al., 2016). The phylogenetic clustering of Bangladeshi GI-24 strains with nephropathogenic Indian-like viruses raises concern that this genotype may contribute to systemic disease manifestations beyond the respiratory tract. Notably, a previous study demonstrated that nephropathogenic IBV can induce renal endoplasmic reticulum stress and trigger apoptosis in chicken kidneys (Chen et al., 2022), supporting the potential of such variants to cause severe renal pathology. Similarly, GI-16 (Q1-like) is recognized as a highly transmissible genotype that has rapidly spread across multiple regions, including Europe and Asia, and has been associated with severe respiratory disease and production losses in both broilers and layers under field conditions. However, confirmation of comparable pathogenicity for the newly detected Bangladeshi strains would require detailed pathological investigations and controlled experimental infection studies. Nevertheless, the emergence of GI-16 in Bangladesh is epidemiologically significant and may contribute to increased outbreak frequency, rapid flock-to-flock transmission, and reduced vaccine effectiveness.

Given that vaccines prepared with Mass-like strains generally provide limited cross-protection against genetically distant IBV lineages, the emergence of GI-24 raises concerns regarding vaccine efficacy and the potential for increased disease severity, particularly renal involvement (Lisowska et al., 2021). Equally significant is the first molecular identification of genotype GI-16 (Q1-like) in Bangladesh. GI-16 was originally reported in China and later spread to Europe, Africa, and South America, where it has been associated with severe respiratory disease, nephritis, and poor vaccine protection (Sjaak de Wit et al., 2011; Rafique et al., 2024). The detection of a GI-16 isolate showing complete nucleotide identity with an Italian field strain (Krapež et al., 2011) highlights the ability of IBV lineages to disseminate over long distances, possibly through international poultry trade or movement of genetic materials.

Pairwise evolutionary distance analysis further demonstrated substantial genetic divergence between circulating field strains and commonly used vaccine strains, even among isolates showing high nucleotide similarity to vaccines. This finding emphasizes that minor variations within the S1 hypervariable regions may significantly affect antigenicity, tissue tropism, and pathogenicity (Shan et al., 2018). Such intra-lineage variability may explain differences in clinical outcomes observed in vaccinated flocks and contributes to apparent vaccine failures reported in the field. This study provides updated molecular insights into the genetic diversity and antigenic evolution of Infectious Bronchitis Virus (IBV) circulating in commercial poultry flocks in Bangladesh. Analysis of the S1 gene revealed the co-circulation of multiple IBV genotypes, including GI-1, GI-24, and GI-16, with distinct patterns of amino acid variation within hypervariable regions (HVRs) known to influence viral tropism, antigenicity, and vaccine efficacy. Although the GI-1 (Mass-type) genotype remains predominant and genetically close to commonly used vaccine strains, the identification of key mutations within immunologically important regions suggests ongoing antigenic drift under vaccine-induced selective pressure.

Several amino acid substitutions were identified at critical positions within HVR1 and HVR2, including residues 38, 64, and 69, which have previously been associated with changes in tissue tropism and virulence. The detection of substitutions such as D38N, G64E, and I69T in multiple GI-1 field strains compared with Ma5 and H120 vaccine strains indicates the possible emergence of vaccine-escape variants. In particular, the G64E substitution observed in strain B131 may have enhanced tissue dissemination, consistent with earlier reports linking this residue to altered pathogenic phenotypes. These findings help explain the continued occurrence of IBV replication and clinical disease in vaccinated flocks.

The emergence of GI-24 and GI-16 genotypes further highlights the dynamic IBV evolutionary landscape in Bangladesh. GI-24 strains shared key substitutions associated with antigenic divergence from vaccine strains, while GI-16 appeared relatively conserved, suggesting more recent introduction or lower immune selection pressure. Differences between Bangladeshi GI-24 strains and the Indian reference strain (IB-MZ-IND), particularly at residue 38, underscore the potential for regional adaptation and independent evolutionary trajectories.

Neutralizing epitope analysis provided additional evidence for limited cross-protection among circulating genotypes. The linear B-cell epitope at positions 87–93 within the S1 protein showed pronounced sequence variability among GI-1, GI-24, and GI-16, whereas the epitope at positions 412–418 remained relatively conserved. Because the 87–93 region plays a critical role in eliciting neutralizing antibodies that block viral attachment, genotype-specific variations in this epitope likely reduce antibody binding and neutralization when heterologous vaccines are used. This antigenic mismatch offers a plausible explanation for vaccine failure observed under field conditions.

Overall, the combined genetic and epitope-based analyses indicate that IBV evolution in Bangladesh is shaped by both vaccine-driven selection and the introduction of novel genotypes. These findings underscore the importance of continuous molecular surveillance, incorporation of antigenically relevant strains into vaccine formulations, and the adoption of heterologous or genotype-matched vaccination strategies to enhance protective immunity against the diverse IBV population currently circulating in the country.

The detection of multiple IBV genotypes, including emerging and globally distributed lineages, reflects the dynamic and evolving nature of IBV in Bangladesh. Given that genotype-specific differences in tissue tropism and virulence have been widely documented, the introduction of GI-24 and GI-16 may further complicate clinical presentation and disease control, particularly in the presence of co-infections and suboptimal vaccination programs. These findings emphasize the importance of continued molecular surveillance coupled with clinical and pathological investigations to determine the pathogenic potential of emerging genotypes and to support evidence-based vaccine selection and control strategies. These findings also highlight the limitations of relying solely on vaccination based on Mass-like strains and reinforce the need for genotype-matched vaccines, specially an additional variant IBV vaccine to broaden the protection or for genotype-matched vaccines, continuous molecular surveillance, and periodic evaluation of vaccination strategies to ensure effective IBV control.

## Conclusions

This study provides the first molecular evidence of emerging IBV genotypes GI-24 and GI-16 in Bangladesh, alongside ongoing circulation of genetically divergent GI-1 strains. Genotype GI-1 (Massachusetts-type) remains the predominant lineage, with evidence of both vaccine-like and field-origin strains circulating under field conditions. Importantly, this study reports the first detection of IBV genotypes GI-24 and GI-16 in Bangladesh, indicating the introduction of genetically distinct and potentially vaccine-evasive lineages into the national poultry population. The observed genetic variation and neutralizing epitope diversity highlight the limitations of current vaccines and support the need for genotype-informed vaccination strategies. These findings support the need for continuous molecular surveillance and evaluation of vaccination strategies to ensure sustainable poultry production.

## Credit authorship contribution statement

**Mohosin Kabir:** Data curation, Formal analysis, Investigation, Methodology, Writing-original draft, Writing-review & editing. **Most Shahana Akter:** Data curation, Formal analysis, Investigation, Methodology, Writing-original draft, Writing-review & editing. **Md. Riabbel Hossain:** Data curation, Formal analysis, Investigation, Methodology, Writing-original draft, Writing-review & editing. **Md. Mohi Uddin:** Data curation, Formal analysis, Investigation, Methodology, Writing-original draft, Writing-review & editing. **Adam Jbenyeni:** Data curation, Formal analysis, Funding acquisition, Writing-review & editing. **Gwenaëlle Dauphin:** Data curation, Formal analysis, Funding acquisition, Writing-review & editing. **Emdadul Haque Chowdhury:** Data curation, Formal analysis, Investigation, Methodology, Writing-original draft, Writing-review & editing, Resources, Supervision, and **Rokshana Parvin:** Conceptualization, Data curation, Formal analysis, Investigation, Methodology, Writing-original draft, Writing-review & editing, Funding acquisition, Resources, Supervision.

## Disclosures

The authors declare that the research was conducted in the absence of any commercial or financial relationships that could be construed as a potential conflict of interest.

## Conflict of interest

The authors declare that the research was conducted in the absence of any commercial or financial relationships.

## Funding

This research was funded by CEVA (Ceva Sante Animale) France.
